# Finishing the finished human chromosome 22 sequence

**DOI:** 10.1186/gb-2008-9-5-r78

**Published:** 2008-05-13

**Authors:** Charlotte G Cole, Owen T McCann, John E Collins, Karen Oliver, David Willey, Susan M Gribble, Fengtang Yang, Karen McLaren, Jane Rogers, Zemin Ning, David M Beare, Ian Dunham

**Affiliations:** 1 The Wellcome Trust Sanger Institute, Wellcome Trust Genome Campus, Hinxton, Cambridge, CB10 1SA, UK; 2 EMBL-European Bioinformatics Institute, Wellcome Trust Genome Campus, Hinxton, Cambridge, CB10 1SD, UK

## Abstract

**Background:**

Although the human genome sequence was declared complete in 2004, the sequence was interrupted by 341 gaps of which 308 lay in an estimated approximately 28 Mb of euchromatin. While these gaps constitute only approximately 1% of the sequence, knowledge of the full complement of human genes and regulatory elements is incomplete without their sequences.

**Results:**

We have used a combination of conventional chromosome walking (aided by the availability of end sequences) in fosmid and bacterial artificial chromosome (BAC) libraries, whole chromosome shotgun sequencing, comparative genome analysis and long PCR to finish 8 of the 11 gaps in the initial chromosome 22 sequence. In addition, we have patched four regions of the initial sequence where the original clones were found to be deleted, or contained a deletion allele of a known gene, with a further 126 kb of new sequence. Over 1.018 Mb of new sequence has been generated to extend into and close the gaps, and we have annotated 16 new or extended gene structures and one pseudogene.

**Conclusion:**

Thus, we have made significant progress to completing the sequence of the euchromatic regions of human chromosome 22 using a combination of detailed approaches. Our experience suggests that substantial work remains to close the outstanding gaps in the human genome sequence.

## Abstract 2

A combination of approaches was used to close 8 of the 11 gaps in the original sequence of human chromosome 22, and to generate a total 1.018 Mb of new sequence.

## Background

The completion of the human genome sequence was the culmination of the 15 year Human Genome Project. The finished sequence contained 2.85 Gb and was estimated to cover 99% of the euchromatin [1]. Thus far the human genome is the only gigabase scale sequence to obtain the necessary high accuracy and near completeness to be published as a 'finished' standard, although the mouse genome is expected to join it soon. However, although significant efforts were made to obtain maximum continuity, the sequence was interrupted by 341 gaps. Of these, 308 gaps covered approximately 28 Mb of euchromatin while the remainder represented the heterochromatin, chiefly centromeres and telomeres. While finishing of the sequence was a major milestone, for completists there remain the nagging questions of whether it is possible to close the gaps, and what lies in those missing sequences.

The process of sequencing the human genome was undertaken using the two approaches of whole genome shotgun [2] and map based clone sequencing [3]. However, only the clone-based strategy, which utilized genome maps and large insert clones, allowed ready application of directed strategies for completion of the sequence [1]. The clone-based strategy involved building contiguous maps of the human chromosomes in large-insert cloning vectors such as bacterial artificial chromosomes (BACs), resolved at a local level by restriction enzyme fingerprinting and ordered and orientated with respect to longer range maps of the genome [4,5]. Individual BACs were then selected from the maps to create a set of clones that minimally covered the genome for sequencing. In the first instance the tilepath BACs were subjected to shotgun sequencing and assembled to produce the draft quality genome sequence. Progressing from this point to a complete sequence by the process of finishing required two major components: first, the maps of clones required completing so that substrates were available for sequencing; and second, the sequence within each clone required refining to the highest level of accuracy with no gaps. Thus, gaps in the genome sequence could be of three kinds. There could be gaps within individual clone sequences where either sequence could not be determined, or there was ambiguity or error in the base call (sequence gaps/errors) [6]. There could be gaps where no clone was available from the map for sequencing, including, but not restricted to, heterochromatic and segmental duplicated regions (map gaps) [7]. The third type of gap would result from a problem with the shotgun assembly or with the underlying BAC, such as a deletion resulting in a false join within the sequence (assembly or insertion/deletion errors) [6,8]. Quality assessments of the finished human genome sequence suggested that sequence gaps/errors were likely to occur at a rate an order of magnitude lower than the rate of human polymorphism (< 1/10 kb), while mis-assembly or insertion/deletion errors were likely to be relatively few [1,6,9], although the precise number remained to be established at all resolutions. In addition, because of the local nature of the sequence assembly for each clone in the clone-based sequence strategy, sequence or mis-assembly gaps were unlikely to affect substantial regions. On the other hand, the number of map gaps was well established and the missing sequence at each gap was known to be on the order of 90 kb on average.

Therefore, to obtain a complete reference human genome sequence requires identification and sequencing of new clones for map gaps and finding and addressing each base ambiguity or error. This would entail a substantial genome curation activity designed to improve the coverage and accuracy of the sequence. In addition, the current reference sequence is a mosaic of clone sequences derived from more than eight individuals. For genes it would be desirable that the allele in the reference sequence is as far as possible representative of the functional form. For instance, the initial chromosome 22 sequence contained a deletion allele of the CYP2D6 gene [10]. Although this form is reasonably common in European populations, it would be preferable to have a complete version as the reference. Furthermore, in certain regions where there is extensive polymorphism, such as the human leukocyte antigen (HLA) locus, there are arguments for maintaining alternative versions of the common haplotype sequences [11].

**Figure 1:**
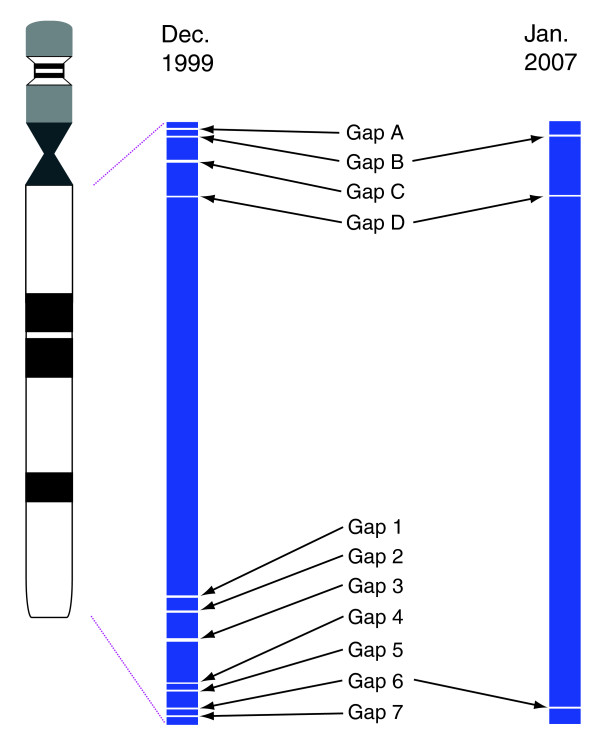
Schematic view of sequence contigs (blue boxes) covering chromosome 22q in [8] (1999, left) and in this report (2008, right). Contigs are drawn approximately to scale and shown in relation to a simple representation of the chromosome 22 ideogram. Gaps are indicated by the arrows, and are labeled according to the terminology used in this report.

Chromosome 22 was the first human chromosome to reach finished sequence standard [8]. On initial publication the sequence comprised 12 contiguous segments spanning 33.4 Mb of 22q (Figure 1) and included known centromeric and subtelomeric heterochromatin repeats at either end. Four of the map gaps were located in 22q11 in regions associated with the segmental duplications involved in low copy repeats (LCRs) on chromosome 22 (LCR22; here referred to as gaps A-D; Figure 1) [8,12-14]. The remaining seven gaps were located in the G+C rich region of 22q13.3 (gaps 1-7; Figure 1) and are not obviously associated with copy number variations (CNVs) in the latest CNV data [15], although CNVs occurring in the gaps would not have been detected.

Since the initial publication we have been working towards closing these gaps, particularly in the 22q13.3 region that was the responsibility of our group in the original chromosome 22 sequencing consortium. Here we report our approaches and progress towards completion of the human chromosome 22 sequence. Our experiences may be pertinent to future efforts to curate the human genome reference sequence.

## Results and discussion

In the following sections we describe our approaches and results towards correction of deletions and closing map gaps on human chromosome 22. The clone library resources used and the information required to decode clone prefixes are provided in Additional data file 1. For reference we have detailed the positions of the gaps and deletions to which we refer on selected genome builds in Additional data files 2 and 3.

### Updating the chromosome 22 sequence to correct deletion alleles and deleted BAC clones

The initial chromosome 22 sequence included a P1 artificial chromosome (PAC) (RP1-257I20, AL021878) containing a common deletion allele of the CYP2D6 gene [10]. In order to represent this gene in a functional form in the reference sequence, we identified from the clone map a RPCI-4 PAC containing a full copy of CYP2D6 (RP4-669P10, BX247885; see Additional data file 1 for details of clone libraries used). This PAC was sequenced and this haplotype, constituting an additional approximately 12 kb of sequence compared to the initial version, was incorporated into the reference sequence assembly.

**Figure 2:**
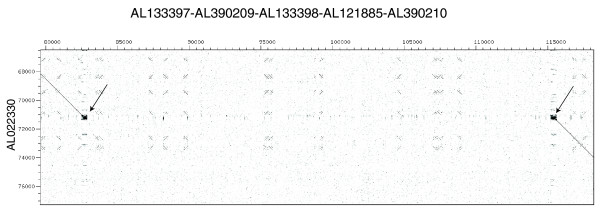
Sequence analysis of deleted BAC CTA-437G10 (AL022330) as originally finished in [8]. A sequence identity dot plot analysis of the revised sequence of this region (x-axis) against the originally submitted sequence (y-axis) generated using dotter [48]. The revised sequence (x-axis) encompasses AL133397.1, AL390209.1, AL133398.2, AL121885.22, AL390210.1 assembled as detailed at [49], and numbered from the start of AL133397.1. Numbering is in base-pairs. Arrows indicate the positions of tandem repeated sequences at the boundary of the deletion in this BAC, with a core region of (AAAG)_46 _at the centromeric (left) side and a core of (AAAG)_68 _at the telomeric (right) side.

Three further cases of large insert clones that contained deletions were identified, either by DNA fiber fluorescence *in situ *hybridization (DNA fiber FISH) or by analysis of human genomic DNA cloned in the WIBR-2 fosmid library. In the first case, during routine physical mapping BAC CTA-437G10 (AL022330) was found by DNA fiber FISH to contain a large (approximately 30 kb) deletion relative to the test DNA sample (Figure S1 in Additional data file 7). It is possible that this deletion represents one allele of an insertion/deletion polymorphism [16]. However, comparison with databases of CNV regions identified within the 270 Haplotype Map (HapMap) samples [17] indicated that the deletion had not been identified as commonly polymorphic [15] and, therefore, an alternative RPCI-1 PAC (RP1-213J1p, represented by AL133397 and AL133398) was identified to span the deletion and sequenced. Thus, it was established that the deletion covered approximately 32 kb and this additional sequence was added into the reference. Comparison of the revised sequence with the original deleted BAC showed that the deletion had occurred between two tandem repeat runs of (AAAG)_n_, suggesting that it may have been mediated via these repeats (Figure 2).

In two further cases, analysis of the distribution of paired end sequences from the WIBR-2 whole genome fosmid library [1] identified two cases where the paired ends were consistently separated by a shorter stretch of DNA sequence than would be expected for this cloning system, or where one of the paired end sequences was missing, indicating the likely presence of a deletion in the reference sequence relative to the haplotypes present in the WIBR-2 fosmid library [1,16]. The presence of deletions in the two RPCI PAC clones contributing to the reference sequence at those points (RP4-633O19, AL022302; RP5-824I19, AL009049) was confirmed by DNA fiber FISH (Figures S2 and S3, respectively, in Additional data file 7). Again, comparison with the known CNV regions did not identify either of these deletions as commonly polymorphic and we chose to identify clones representing the non-deleted forms in both cases for sequencing and inclusion in the reference sequence (G248P-80423F1, BX470187; CITF22-91B8, BX936361 and CITF22-37A6, BX927085; Figures S11 and S12 in Additional data file 7). This sequencing generated an additional approximately 34 kb and approximately 49 kb in the reference sequence covering the two deleted regions, respectively. Comparison of the revised sequences with the original deleted clones showed that the deletions in these cases occurred within repetitive sequences (between two copies of MLT1L repeats for RP4-633O19 (AL022302) and between an AluYa5 and a MLT2C1 repeat in RP5-824I19 (AL009049)).

Analysis of the 126 kb of additional sequence generated to cover the four deletions identified that, in addition to CYP2D6, only a single short predicted ribosomal protein gene (G248P-80423F1.C22.1 in BX470187) had previously been missed due to deletion within the bacterial clones used for the initial reference sequence. However, since there is no mRNA or expressed sequence tag mapping with high sequence identity to this gene, it is likely to represent a pseudogene (definitions as in [18]).

### Closing map gaps in the chromosome 22 sequence

During the initial sequencing of chromosome 22q, the construction of physical maps was divided by region between the different members of the consortium [8]. Our initial map gap closure efforts focused on the seven gaps in 22q13, which are numbered gaps 1-7 from centromere to telomere (Figure 1; Figures S4-S10 in Additional data file 7; see Additional data files 2 and 3 for positions of gaps on selected genome builds). In two cases initial mapping had identified clones that, while containing sequences on either side of the gaps, were deleted when analyzed by DNA fiber FISH analysis (data not shown).

The initial mapping process had involved screening of at least 20 genome equivalents of large insert clone libraries (BAC or PAC) and cosmids or fosmids generated from flow sorted chromosomes (see Additional data file 1 for clone libraries used). As a first step to closing the gaps, we identified sequences at the edges of each gap to develop into sequence tagged sites (STSs) and re-screened these libraries. In the case of the flow sorted chromosome 22 fosmid library (CITF22) [19,20] it was clear that additional clones that extended into the gaps had been missed due to false negatives in initial screens in some cases. These clones were incorporated into the maps by restriction enzyme fingerprinting, extension confirmed by DNA fiber FISH and then appropriate clones were chosen to generate sequence extending into the gaps. There subsequently followed further rounds of chromosome walking by developing STSs from the ends of each fosmid and re-screening the CITF22 fosmid library (Figures S4-S13 in Additional data file 7; Additional data file 4). With this approach we were able to add additional sequence to extend each gap, ultimately contributing approximately 307 kb of the total additional sequence generated for gaps 1-7 in 22q13 (42.4 %; Table 1). In addition, we identified two chromosome 22 specific STSs (AFMb040xdl and WNT7B) for which we had previously been able to identify BAC clones, but had been unable to incorporate these clones into the physical maps at the required stringency of overlap. Re-examination of these data followed by sequencing of two BACs (RP11-435J19, BX511035; RP11-398F12, AL929387), together with an additional PAC (RP6-109B7, AL121672) that had not been finished previously, placed them as extensions into gap 3, adding an additional 137 kb to the total sequence (18.9 %). As the identification of novel clones from these resources became exhausted, an additional source of fosmid clones became available in the form of the WIBR-2 whole genome library with systematic determination of paired end sequences (Whitehead Institute random genomic fosmid library WIBR-2; Additional data file 1) [1]. The availability of end sequences from this library on the NCBI Trace Archive [21] allowed rapid sequence similarity searching to identify fosmids that extended into the unsequenced gaps. The WIBR-2 fosmids contributed an additional approximately 151 kb (20.9 %) of new sequence. In certain cases we were able to alternate chromosome walking between the CITF22 and WIBR-2 fosmid libraries, for instance while closing gap 5 (Figure S8 in Additional data file 7). Although these walking approaches generated additional chromosome 22 sequence, they were able to close only one of the seven gaps.

**Table 1:** Sources of additional sequence for gaps 1-7 in human chromosome 22q13

Source of sequence	Number	Sequence contributed (kb)
Flow sorted chromosome 22 fosmids	14	307
Whole genome fosmids	6	151
BAC/PAC	3	137
PCR products	28	129

Note that due to the arbitrary nature of the positions of clipping of submitted sequences, and their contribution to the final golden path, the new sequence contribution numbers are, of necessity, approximated.

**Figure 3:**
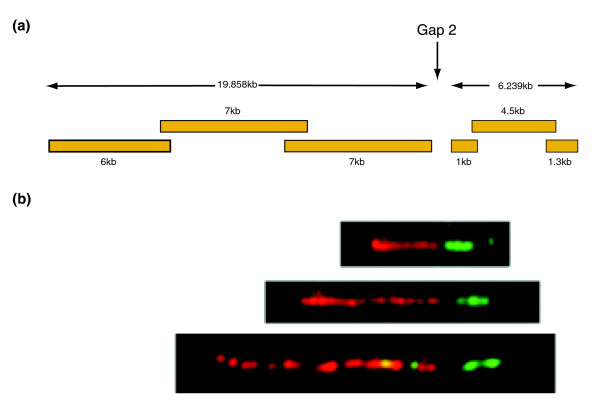
DNA fiber FISH using pools of long PCR products at gap 2 prior to final closure by long PCR. **(a) **Schematic representation of the three long PCR products from either side of the gap that were labeled and hybridized to DNA fibers. See Figure S5 in Additional data file 7 for identities. Note that non-repetitive sequence represented 71% of 19,858 kb on the left-hand side of the gap and only 50% of 6,239 kb on the right-hand side, as determined by Repeatmasker. **(b) **Three example DNA fibers showing detection by FISH of hybridized centromeric and telomeric PCR product pools in red (Texas Red) and green (FITC), respectively. Estimation from the DNA fiber FISH images suggested that the remaining gap was 2-3 kb in size and this was closed by the PCR product c658c926rcL (sequence CU210860).

In order to make further progress towards completion, we adopted an alternative approach of utilizing long PCR between known sequences at the edges of the gaps, or internal to the gaps. Internal sequence was identified from four sources. First, we searched the chimpanzee genome sequence assembly [22] for orthologous sequences matching the human gap boundaries, and identified chimpanzee contigs extending into the gaps. Second, as part of the process of generating sequences for identification of single nucleotide polymorphisms during the HapMap project, six chromosome equivalents of shotgun sequence reads had been generated from plasmid libraries of flow sorted chromosome 22 from three individuals (whole chromosome shotgun sequence (WCS)) [23]. Nearly 890,000 paired end shotgun sequence reads produced from these plasmids and the CITF22 flow sorted chromosome fosmid library were used to produce a scaffold assembly (see Materials and methods). These shotgun sequence reads were assembled (see Materials and methods for details; assembly available at [24]) and the assembly compared to the existing finished sequence. Contigs and scaffolds that extended into the gaps were identified. Third, in one case prior gene annotation [18] indicated the presence of a gene that was only partially contained within existing sequence (gap 1, dJ345P10.c22.4.mRNA). Examination of the cDNA (AK023424) and expressed sequence tag sequences was used to identify putative exons internal to the gap. Finally, Celera shotgun sequence [2,25] was identified that overlapped with chimp scaffold sequence and extended further into the gap. Sequence from each of these sources predicted to lie internal to the gaps was used together with the known sequence at the edges of gaps to design long PCR oligonucleotide primer pairs that might amplify across the missing sequence (Additional data file 5). Wherever possible, long PCR primers were designed so that the PCR product would encompass the shortest possible span of missing DNA. In addition, where a contig from chimpanzee or flow sorted shotgun sequence extended within a gap, we also aimed to amplify this sequence by long PCR from human DNA in order to cover gaps within the contigs/scaffolds to obtain human sequence in the case of chimp and to obtain independent verification of the unfinished WCS sequence. In one case additional long PCR primers were generated from newly sequenced long PCR products. Long PCR products from a pool of human DNAs (Roche high molecular weight human DNA; see Materials and methods) were obtained in the size range 1.3-20 kb (Figures S4-S7, S9, and S10 in Additional data file 7; Additional data file 5). These products were sub-cloned into plasmid vectors and sequenced (see Materials and methods). One gap, gap 2, was difficult to amplify across in the final stages of the PCR gap closure, despite expectations that it was < 5 kb. To allow precise refinement of long-PCR conditions based on an accurate estimate of gap size, we performed two-color DNA fiber FISH by labeling long PCR products on either side of the gap, totaling approximately 20 kb and approximately 6.2 kb, respectively (Figure 3). The remaining gap was thus estimated at 2-3 kb and was then successfully closed using modified PCR conditions.

At the end of several iterations of these strategies we succeeded in closing six of the seven gaps in 22q13 (Figure 1; Figures S4-S10 in Additional data file 7). The one remaining gap, gap 6, was found to be bordered by repetitive sequences sufficient to prevent further long PCR and exact size determination by fiber FISH. However, a fiber FISH estimate from the flanking clones, together with the addition of 10.5 kb of sequence by long PCR, suggests that the sequence remaining to be determined is less than 15 kb. In total, we generated 724 kb of new sequence to close and extend into these gaps, and the final sequences were incorporated into the latest version of the human chromosome 22 reference sequence (Chr_22 release 4 [26], to be incorporated into the next release of the human genome reference sequence: HGRC 37).

During the period of this work, one further gap in 22q11 was closed by chromosome walking in BACs (Figure 1; gap C, AC016026 and AC016027; BA Roe, personal communication). Additional sequence was also produced by the Roe group to shorten the gap between original tile path clones AC007663 and AC007731 (gap D, AC058790, AC024070 and AC011718). From analysis of end sequences from the WIBR-2 whole genome fosmid library, we have identified a single fosmid clone to close the most centromeric gap in 22q11 (gap A, G248P-1690I13, CR545463; Figure S13 in Additional data file 7).

### Quality control and analysis of new chromosome 22 sequences

Although the long PCR systems we used utilize proofreading polymerases in combination with *Taq *polymerase, they are still susceptible to base incorporation errors when compared to sequencing from bacterial clones. In addition, there is also the possibility that rearrangements could be introduced and propagated during the PCR process. To minimize the effect of base incorporation errors, we constructed libraries of each uncloned long PCR product directly in the sequencing vector and sequenced up to 20-fold coverage. In addition, after generating finished sequence across the gaps, we analyzed that sequence in several ways to check for error. First we examined regions of overlap between sequence templates, which, therefore, had been sequenced twice from independent cloning events, either from long PCR product and bacterial clone (BAC, PAC or fosmid) or from two independent long PCR products. In the case of overlaps between long PCR derived sequences and bacterial clones, 11 base differences were identified out of 11,676 bp examined. Comparing overlapping long PCR products identified 27 base differences in 37,810 bp of sequence overlap. Both these base difference rates are consistent with the expected rates of variation due to sequence polymorphism between any two human genomes [27-29]. When we analyzed short PCR products representing 25 out of these 27 differences from 6 different individuals by single pass sequencing, only 2 were not either single nucleotide polymorphisms (19 out of 25) or short polymorphic runs of Ts (4 out of 25). Assuming these two base changes were sequencing errors and not rare polymorphisms, these data would give an error rate typical of finished quality sequence (2 out of 37,810 bp) [6,9]. We also sought additional confirmation of the quality of sequence produced from long PCR products by designing oligonucleotides from the long PCR sequence to generate an overlapping path of shorter PCR products (< 1,200 bp). Comparison of single pass sequencing of 219 PCR fragments with 84,546 bp of genomic sequence confirmed the integrity of the sequence in all cases. Base by base examination of 13,239 bp of single pass sequence from one region (gap 3) identified only 2 possible errors, both of which were single base length difference in short repeat runs (poly T and poly CA), that could potentially be polymorphic, but which are also known 'difficult' sequences in single pass PCR sequencing. From these analyses we conclude that the sequence derived from the long PCR products is of a similar standard to conventional finished sequence from bacterial clones and the process was successful in closing recalcitrant gaps.

We subjected the new sequence from gaps 1-7 and the four deleted regions (approximately 820 kb) to sequence analysis and gene annotation using our standard approach [18,30] (additional annotation available at [26]). In total, 14 new coding genes, 2 non-coding genes (without an open reading frame of > 300 bp) and one pseudogene were annotated on to the chromosome 22 sequence including CYP2D6. No high identity matches (≥95% identity) were found to known human microRNA precursors. Seven of these annotations involved extension of genes that were partially annotated previously [18] and extended into the previous gaps. One of the annotated genes is a partial structure. This gene annotation includes an additional 95 exons contributing 23,872 bp beyond the annotation described in [18], which included some annotations on added sequence. Therefore, addition of 820 kb (2.3%) of new chromosome 22 sequence within the previous gaps has increased the gene annotation by 1.6-1.8% (by base content or exon count, respectively).

In order to try to understand whether particular phenomena underlie the persistent recalcitrance of certain genomic regions to sequencing, we re-examined the features of the sequences that surround and now close the gaps. At the telomeric boundary of the unclosed gap, gap 6, there is an 11.2 kb sequence consisting of 87% repetitive sequences and a GC-rich (72%) unique segment that has made it impossible with current sequencing technology to walk further into this gap. Analysis of the GC content of the new sequences revealed that five of the gaps have significantly higher GC content (mean GC ranging from 54.5-57.5%) than the approximately 7.3 Mb of 22q13 (mean GC 51%) in which they reside (*p *< 10^-4^, Wilcoxon rank sum test, 1 kb windows; Figure S14 in Additional data file 7). The two exceptions are gap 5, which was closed entirely with fosmids and has a GC content similar to the mean for 22q13 (51%), and gap 1, with a GC content that is higher (mean 54%) but differs less significantly from the surrounding sequence (*p *< 0.02). Given that chromosome 22 overall and 22q13 particularly have high GC content compared with the rest of the genome, it is clear that these sequences are unusual. Further analysis by sequence dot plot showed that several of the gaps also contain substantial simple tandem repeat sequences (Figure S15 in Additional data file 7). Comparing the density of tandem repeat bases per kilobase in 25 kb windows between the gap sequences and all chromosome 22 sequence indicated that the gaps are significantly enriched in tandem repeats (*p *= 6.756e^-10^, Wilcoxon rank sum test, 25 kb windows). Taken together with the observation of the role of tandem repeats in the deletion of BAC CTA-437G10 (AL022330), it seems plausible that further investigation of the role of tandem repeats in these problem sequences is warranted.

## Conclusion

Since the essential completion of the sequence of chromosome 22, 8 out of the 11 gaps have been closed by conventional mapping combined with novel PCR-based approaches. In total we generated 1.018 Mb of new sequence to extend into and close these gaps, and the final sequences were incorporated into the latest version of the human chromosome 22 reference sequence; 95 additional exons were identified for 16 genes and one pseudogene, contributing an additional approximately 1.6 % of gene annotation. In a parallel effort Bovee *et al*. [31] have used fosmid libraries generated from the DNA of multiple individuals to close 26 gaps across the genome, including identification of two fosmids across two of the gaps addressed here (gaps 2 and 7, referred to as 22_05 and 22_10 in Table 1 of [31]). These findings have confirmed the sequence additions reported here and, indeed utilized and built on the intermediate assemblies generated and released as part of this project. However, both studies illustrate the likely success of approaches that can be taken towards closing the remaining gaps in the human sequence. In the Bovee *et al*. study, a high throughput approach using new fosmid libraries was successful for 10% of the remaining gaps in the human genome. In this study we took a more exhaustive approach including fosmid libraries but also utilizing all available sources of sequence information in a PCR based strategy. In our case we were able to close 8 (72.7 %) out of the 11 remaining gaps on chromosome 22 studied, and all bar one of the gaps not associated with the LCR22 segmental duplications. Thus, it seems likely that the majority of gaps in the human genome will be tractable to closure strategies, some via initial high throughput approaches and the remainder by more detailed analysis. However, although high throughput approaches can be rapid, the more detailed analysis we have undertaken is not, as it involves multiple cycles of experimental work and decision-making, plus a degree of trial and error when amplifying by long PCR. Extending this approach genome-wide would require substantial investment in experienced genome mappers, unless the processes can be substantially streamlined. Alternatively, combining surface based or solution capture methods utilizing the sequences at the edge of gaps with high throughput sequencing (see, for instance, [32]) of the enriched sequence might provide a way into outstanding gaps. Hence, future progress on closure of gaps is likely to depend on the level of motivation that exists for the project or development of additional technologies.

In addition to closure of gaps, we also added 126 kb of sequence to patch 4 regions of the initial chromosome 22 sequence that had been identified as harboring deletions either as a result of polymorphisms, or clone artifacts. It is clear that providing these problem regions can be efficiently identified, it is possible to provide fixes. In these cases, one was identified from study of a known gene, two from analyzing the mapping of WIBR-2 fosmid end sequences and the other during routine mapping via DNA fiber FISH. Both fosmid end sequence mapping and DNA fiber FISH methods are unlikely to identify problems below 5-10 kb in size. Hence, small insertion/deletion problems (or polymorphisms) such as retrotransposon insertions will not be caught. In the future large-scale high throughput sequencing of many human genomes may provide resources that can be used to identify such insertion/deletion events. The question then will be whether it makes sense to maintain a single human reference sequence, and if so, to what criteria that reference should conform (for example, single haplotype, functional haplotypes, longest genome). The current reference sequence is a mosaic derived from more than eight individuals. However, approximately 70% of the reference assembly originated from a library of a single individual (the RPCI-11 BAC library). While it might be attractive to migrate the reference sequence towards defined haplotypes, this would be substantial additional work and in practice most regions are already likely to represent relatively common haplotypes because of the nature of human polymorphism [27,33,34].

Similarly, the availability of additional sequence of this type may provide sequence within current gaps. However, it is important to remember that in addition to the well mapped gaps residing in the 'euchromatin' of the human genome, there are many megabases of unknown human hetereochromatin still to be sequenced, including centromeres, the p arms of the acrocentric chromosomes, other heterochromatic blocks and some segmentally duplicated regions. We have provided some sequence for chromosome 22p, both as whole chromosome assembly [35] and as isolated BAC and fosmid clones (unpublished data list of chromosome 22p accessions in Additional data file 6). Placing new sequence into the increasingly small complement of euchromatic gaps will require substantial effort to verify its location, possibly using traditional mapping tools such as flow sorted chromosomes and somatic cell hybrids. In our opinion much mapping work remains to be done before we will see the complete human genome sequence.

## Materials and methods

### Bacterial clone library screening and contig construction

Conventional mapping of bacterial clones, PCR, hybridization and building of contigs was performed by standard protocols as described in [4,36,37]. Clone libraries used in this work are detailed in Additional data file 1.

### Sequencing

Genomic sequencing of bacterial clones and sequencing of short PCR products was performed as previously described [38-40].

Long PCR products were sequenced using small insert libraries prepared from gel-purified PCR products [38]. Long PCR products were purified on low melting temperature agarose gels, sonicated and end repaired with mung bean nuclease. Small inserts of 300-500 bp and 500-800 bp were subcloned into pUC18 for sequencing and assembled using the PHRAP algorithm [38].

Sequence assemblies to form a golden path (agp) across new sequence and a whole chromosome sequence was performed as described in [41]. Additional data files 2 and 3 give details of positions of the gaps and deletions referred to here in multiple representative genome assemblies. Annotation of sequence was as described previously [30]. Tandem repeats were identified using tandem repeat finder [42]. Additional sequence processing and analysis was performed using custom perl scripts and R [43]. The agp file describing the chromosome 22 sequence assembly, the sequence in fasta format and the new and extended annotations in general feature format (GFF) are available at [26].

### Long PCR

Long PCR primer pairs were designed using primer 3 [44] with specifications as recommended by the Roche Expand 20 kb+ kit (catalogue number 1-811-002, Roche Diagnostics Ltd. Burgess Hill, UK). Most long-PCR amplifications were performed using Roche high molecular weight DNA (catalogue number 1-691-112), but in regions of repeated DNA sequences that proved difficult to assemble, additional PCR reactions from single individuals were amplified and sequenced. Long PCR was performed using either Roche Expand 20 kb+ PCR system (initially Pwo/Taq enzyme combination prior to introduction of the Tgo/Taq version), Roche Expand Long Template-PCR System (catalogue number 1-681-842) or Novagen KOD Hot Start DNA polymerase kit (catalogue number 71086-4, Novagen (Merck Biosciences Ltd) Nottingham, UK). PCR conditions were as recommended by the manufacturers with the optional addition of between 1% and 6% dimethyl sulphoxide for some reactions. DNA (200-240 ng) was amplified in thin walled PCR tubes in 50 μl reactions and at annealing temperatures of 62-65°C for 35 cycles. c658c926rcL (gap 2 final closure) included 1:1 7-deaza dGTP:dGTP, 3% dimethyl sulphoxide and modified PCR cycle temperatures of 65°C annealing, 72°C extension (first two cycles) and 95°C denaturing.

### Standard PCR

Standard PCR primers were designed using primer 3 [44] and amplified as described previously [39]. Primer design specifications were adjusted to take into account regions of high GC content and denaturing times/temperatures were increased. To generate the short PCR tiling paths and analyze possible polymorphisms, DNA samples from six individuals were amplified (three HapMap samples, NA07340, NA12873 and NA17119, plus NA06990, NA10847 and NA12873) in addition to the mixed Roche DNA employed to generate the long PCR products.

### Fiber FISH

Fiber FISH and digital imaging essentially followed the procedure as described previously [45], with a slight modification in the preparation of DNA probes when PCR products were used. Briefly, in the case of gap 2 (Figure 3) three PCR products from each side of the gap were selected for FISH. PCR products were first cleaned using a GenElute^® ^PCR Clean-Up kit (Sigma-Aldrich, Gillingham, Dorset, UK) and quantified on a NanoDrop^® ^ND-1000 spectrophotometer (Labtech International, Ringmer, East Sussex, UK). The cleaned PCR products from each side of the gap were pooled at equal molarity for each PCR product, and then labeled using either the Biotin-Nick Translation Mix and DIG-Nick Translation Mix (Roche Diagnostics, Burgess Hill, West Sussex, UK). Biotin-labeled probe was detected with Texas Red^® ^conjugated avidin (Molecular Probes, Eugene, Oregon, USA). DIG-labeled probe with monocolonal mouse anti-dig antibody (Sigma-Aldrich) and FITC-conjugated goat anti-mouse antibody (Vector Laboratories, Orton Southgate, Peterborough, UK).

### Shotgun sequence assembly

In the HapMap project, DNA samples of three individuals were flow sorted for chromosome 22 and 889,608 shotgun reads (approximately 2× read coverage for each individual) were generated mainly from plasmid libraries with an averaged insert size of 2.5 Kb [23]. The WCS dataset also included approximately 60,000 fosmid end sequences, separated by around 35- 40 kb, that were generated from a flow sorted chromosome 22 library [20]. To get a global sequence view of the whole of chromosome 22 and close possible gaps in 22q, a strategy was pursued to produce a shotgun assembly using all the HapMap reads including plasmids and fosmid sequences. The Phusion assembler was used to assemble the chromosome using WCS reads [46]. Aligning the contigs and scaffolds against the existing finished sequence, it was possible to identify the gaps where the reference sequence does not have coverage. The WCS assembly and HapMap reads are available for download at [47]. This was the main approach used for identifying sequence within gaps. In addition, to effectively separate novel contig sequences including gaps and 22p from the whole chromosome shotgun, we pursued a second strategy: using 22q finished sequences to guide the WCS assembly. Finished clone sequences were shredded into fragments with a read length of 1,000 bp and a paired insert size of 40,000 bp. The shredded reads accounted for about 2× coverage over 22q. Assigning the shredded reads back to 22q finished sequences, it was possible to build an assembly by removing those contigs that can be firmly placed on existing 22q sequence. It was expected that the assembly with mixed shredded data could be better than the pure WCS assembly in which the shotgun reads were from three individuals, and might place sequences extending immediately into the gaps at the borders of firmly mapped contigs. We have used this '22p assembly' (including any non-mapped sequence from 22p, 22 cen and 22q gaps) as a source of probes to screen for BAC clones in 22p (Additional data file 6). We have placed the mixed and 22p assemblies (including any non-mapped sequence) at [35] for any future applications.

## Abbreviations

BAC, bacterial artificial chromosome; CNV, copy number variation; FISH, fluorescence *in situ *hybridization; HapMap, haplotype map; LCR, low copy repeat; PAC, P1 artificial chromosome; STS, sequence tagged site; WCS, whole chromosome shotgun sequence.

## Authors' contributions

CGC carried out long PCR gap filling and sequence validation experiments and analysis. OTM carried out bacterial clone library screening, map building by restriction enzyme fingerprinting, long PCR and fiber FISH. JEC and DMB carried out gene annotation of the novel sequences. KO, DW, KM, and JR were responsible for library preparation and DNA sequencing of gap clones and PCR products. SMG and FY supervised and conducted DNA fiber FISH experiments. DMB carried out sequence analysis, sequence assemblies and management of AGP and tpf infrastructure. ID designed and supervised the project, conducted sequence analysis of gap sequences and assembled figures and tables. ID and CGC prepared the manuscript. All authors have read and approved the final manuscript.

## Additional data files

The following additional data are available with the online version of this paper. Additional data file 1 is a table listing the clone libraries used in this work. Additional data file 2 is a table listing the coordinates of the gaps and deletions addressed in this work on numerous genome builds. In addition, this file contains the tile path file (tpf) specification for chromosome 22. Additional data file 3 contains the Additional data file 1 table and the tpf file in tab delimited text format. Additional data file 4 is a table listing the STSs used in clone library screening as referred to in Figures S4-S13. Additional data file 5 is a table listing the long PCR products generated. Additional data file 6 is a table listing clones identified as mapping to human chromosome 22p or 22cen. Additional data file 7 contains Figures S1-S15.

## Supplementary Material

Additional data file 1 Clone libraries used in this work. Click here for file

Additional data file 2 Coordinates of the gaps and deletions addressed in this work on numerous genome builds and the tile path file specification for chromosome 22. Click here for file

Additional data file 3 The table and tpf file from Additional data file 1 in tab delimited text format. Click here for file

Additional data file 4 STSs used in clone library screening as referred to in Figures S4-S13. Click here for file

Additional data file 5 Long PCR products generated. Click here for file

Additional data file 6 Clones identified as mapping to human chromosome 22p or 22cen. Click here for file

Additional data file 7 Figures S1-S15. Click here for file
