# Supplementary material for: Finishing the finished human chromosome 22 sequence
Source: Genome Biol. 2008 May 13;9(5):R78. doi: 10.1186/gb-2008-9-5-r78 (PMC2441464; doi:10.1186/gb-2008-9-5-r78)
Supplement: Additional data file 1 — Clone libraries used in this work. [file gb-2008-9-5-r78-S1.doc]

**Table S1**

Clone libraries used in this work.

| **Library Name** | **Abbreviation** | **Description** | **Vector Type** | **Identifier Prefix** | **Reference** |
| --- | --- | --- | --- | --- | --- |
| RPCI Human PAC library  RPCI Human BAC library  Caltech Human BAC library  LL22NCO3 "N”  Sanger Institute flow-sorted chromosome 22 cosmid.    Caltech flow-sorted chromosome 22 fosmid,  Whitehead whole genome fosmid | RPCI-1, -3, -4, -5, -6  RPCI-11, RPCI-13  CIT978SK, CIT-HSP  LL22NCO3  SC22cB  CITF22  WIBR-2 | Human male (RPCI-1, 3, 4, AND 5) or female (RPCI-6), partially MboI PAC library. See <http://bacpac.chori.org/humanmalepac.htm>.  Human male (RPCI-11) or female (RPCI-13) BAC library. See <http://bacpac.chori.org/hmale11.htm>, <http://bacpac.chori.org/femalehum13.htm>.  Human male BAC library (segments A1, A2, B, C).  Lawrence Livermore “N” Flow-sorted chromosome 22 cosmid library  Sanger Institute flow-sorted chromosome 22 cosmid library.  Caltech flow-sorted chromosome 22 fosmid library  Whitehead/Broad Institute whole genome human fosmid library. | PAC  BAC  BAC  Cosmid  Cosmid  Fosmid  Fosmid | RP1-, RP3-, RP4-, RP5-, RP6-  RP11-, RP13-  CTA-, CTB-, CTC-,  LL22NC03-  cB  CITF22-  G248P- | Ioannou et al. Nat Genet. 6:84-9 (1994).  Osoegawa et al. Genome Res. 11:483-496 (2001).  Kim et al. Genomics 34, 213-218 (1996).  de Jong et al. Cytogenet. Cell Genet. 51:985 (1989).  N/A  Kim et al. Genet Anal 12:81-84 (1995).  International Human Genome Sequencing Consortium. Nature 431:931-945 (2004). |

Notes:

RPCI-1, -3, -4, -5, -6, -11, -13, WIBR-2 libraries are available from Children's Hospital Oakland Research Institute at http://bacpac.chori.org/.

CIT978SK library segments available from Open Biosystems at <http://www.openbiosystems.com/>.

See also <http://www.sanger.ac.uk/HGP/methods/mapping/info/lib-details.shtml>.

Clones not available from the sources above can be requested from geneservice at <http://www.geneservice.co.uk/>.
