# Supplementary material for: Finishing the finished human chromosome 22 sequence
Source: Genome Biol. 2008 May 13;9(5):R78. doi: 10.1186/gb-2008-9-5-r78 (PMC2441464; doi:10.1186/gb-2008-9-5-r78)
Supplement: Additional data file 4 — STSs used in clone library screening as referred to in Figures S4-S13. [file gb-2008-9-5-r78-S4.doc]

**Table S3**

STSs used in library screening as referred to in Figures S4-S13. Note accession numbers are given for parent sequences except in the case where the STS was derived from an end sequence of a clone, in which case the original internal identifier for the end sequence is given.

| **STS No.** | **STS Name** | **Oligo 1** | **Oligo 2** | **Length** | **Parent**  **Sequence** | **End of Clone** |
| --- | --- | --- | --- | --- | --- | --- |
| stSG17510 | stAFMb040xd1 | CTTTGGGGAAGCAGTGAG | GGATTCTGTCTATTTGTTATGGTC | 216 | Z53191 | NA |
| stSG20665 | stdJ32I10T7 | CAGAGGCAGCAGAGGAGC | CCTGAAGGAGGGAGAGCTTT | 131 | RP1-32I10T7 | RP1-32I10 |
| stSG20701 | stcN13B1T7 | TCTGTGCATTGTGGGATGTT | CAGGGCAGCAGCTTAACTG | 150 | LL22NC03-13B1T7 | LL22NC03-13B1 |
| stSG35834 | stdJ1033E15T7 | AAGGCTTATGAGCAGCCAGA | CACACACACACAAAGGGAATG | 162 | dJ1033E15T7 | dJ1033E15 |
| stSG43737 | stWNT7B | GAAGGAGAAGTACAACGCGG | GTCCTCCTCGCAGTAGTTGG | 160 | AF416743 | NA |
| stSG93171 | stdJ185D5.1 | CATCCATCTGCCCCTCAC | AATGAGCTCTGGAAAGAAGGC | 148 | AL118498 | NA |
| stSG93173 | stdJ185D5.4 | AACAAAGGTGACACAAGTGTGG | ATTACGGGACAGAGACGCC | 120 | AL118498 | NA |
| stSG93207 | ststdJ185D5.A | CCAGTCTGTGGGTTCTCTTTT | ATCTACATGACCTTGGGTTTGG | 165 | RP1-185D5A | RP1-185D5 |
| stSG96244 | stdJ619N21_3877 | CACTAGGGGATTTGGGGTTT | CAGCCAGGAGAACAGAGACC | 444 | AL078607 | NA |
| stSG119114 | stbA262A13L | GCGAAGTGTCCACAGAACAA | ATATTTGGAGAATGGCGCAG | 299 | RP11-262A13L | RP11-262A13 |
| stSG119115 | stdJ579N16A | GTCTCACGTTTGTGTGGTGG | TTGCAGCCTTTCTTTCCAGT | 248 | RP4-579N16A | RP4-579N16 |
| stSG133679 | stfF4H11B | ACTGAAACGCAGGCCTCTAA | TTATCCTCCCAGGAGGGC | 185 | CITF22-4H11B | CITF22-4H11 |
| stSG145614 | stbA494O16A | ATAGCAGCAAAAGGCTGGAA | CAGACTTGGTGAGGGTTGGT | 149 | RP11-494O16A | RP11-494O16 |
| stSG145639 | stdJ619N21B | CCCAGTACCCATTGCACTG | GACAAGTAGGTGGCATACCCA | 163 | RP4-619N21B | RP4-619N21 |
| stSG145676 | stdJ619N21A | ATCCTGCTCAGTGGTCTGCT | GACACGGGTGAAAGTCGATT | 128 | RP4-619N21A | RP4-619N21 |
| stSG158873 | stSG158873 | TTCAAGCAGAAGTCCCTCTAGG | CCTCCAGGGTGACCTCCT | 122 | Z85994 | RP1-32I10 |
| stSG160465 | stSG160465 | TCCTTGCCTAGTGGCACTG | TCCTCCCATAATTGAGCCTG | 123 | CITF22-53E7.sp6 | CITF22-53E7 |
| stSG363199 | stSG363199 | TCTGCTTGCAGAACGCTG | CCTCCTTTTCTCTGTGCCTG | 187 | AL096843 | NA |
| stSG363200 | stSG363200 | TCTCCCCTGTCAAAACTTGG | AAAGCATCCGGCAAAATTC | 170 | AL096843 | NA |
