# Supplementary material for: Finishing the finished human chromosome 22 sequence
Source: Genome Biol. 2008 May 13;9(5):R78. doi: 10.1186/gb-2008-9-5-r78 (PMC2441464; doi:10.1186/gb-2008-9-5-r78)
Supplement: Additional data file 5 — Long PCR products generated. [file gb-2008-9-5-r78-S5.doc]

**Table S4. Summary of Long PCR Products Used to Close Sequence Gaps on Human Chromosome 22.**

| **Sanger LPCR Product name** | **Accession** | **Primer_1 sequence** | **Primer_1 Sequence Source** | **Primer_1 Source Code** | **Primer_2 Sequence** | **Primer_2 Sequence Source** | **Primer_**  **2 Source Code** |
| --- | --- | --- | --- | --- | --- | --- | --- |
| c005500511 | BX890592 | AGTGACAGTCCCTGTCTTATGCTGG | AL049708 (pryM763A3a) | YE | ACCACCACTGTGAAAATGCCTTCTA | 9999.c005500511.Contig1 | SG |
| c0511c0695L | CU137653 | ACGGGACTCGAAGGTTACCATAAGA | 9999.c005500511.Contig1 | SG | GGAAAGCAGAGAAAAGAGACCAGGA | 9999.c008600695.Contig1 | SG |
| c008600695rc | CR377231 | GCTTTACATCAGCAGGAGGGAGTTT | 9999.c008600695.Contig1 | SG | CATGCAGGATGTTGTGATCATCTTC | 9999.c008600695.Contig1 | SG |
| FW80414D10l | BX546033 | ATCTCACTCCTGGGCTGTGTATCAG | 9999.c008600695.Contig1 | SG | TAAGACCTGAGGGGTTCAGCTTTTC | AL929420 (CITF22-123F2) | FO |
| c017600455L | CR933566 | GGTAGGTGCTGAATAAATGTCTGCCG | 9999.c017600455.Contig1 | SG | TCTGGGTTCTAAGTCTGGTGTCAGG | 9999.c017600455.Contig1 | SG |
| c455c658L | CR936488 | TTATAACAAACCAAAGCACCAACGC | 9999.c017600455.Contig1 | SG | GTCCGCCTCTGTATTCTCACTTTGA | 9999.c009500658.Contig1 | SG |
| c009500658L | CR936470 | ATCATCCCTTCATTCTCAGCAACCT | 9999.c009500658.Contig1 | SG | GAAGCTCATTAGTGCCCGTGGTT | 9999.c009500658.Contig1 | SG |
| c658c926rcL | CU210860 | AGGGACTTCTCATCAGTCTGGGTCT | 9999.c0095c00658.Contig1 | SG | ATCTCTTTACACCAGAGGTCTCCCG | 9999.c010402926.Contig1 | SG |
| c926rcL | CT009695 | ACCCTTCACATGAGACAAGCTC | 9999.c010402926.Contig1 | SG | CACTGAAGACAGGAAGGGATG | 9999.c010402926.Contig1 | SG |
| chimpc702L | CR954960 | CCCTTTCAAAGATAAGAGGGAGGCT | AACZ02196519^1^ | CH | GCTTTAAATTTAGCTCCCTCCCCAG | 9999.c024501702.Contig1 | SG |
| c024501702 | BX890582 | GTGAGACGGAGCATGTAAGCATGTAA | 9999.c024501702.Contig1 | SG | TGCCTGCACACAGAGCAACTACTAC | AL929500 (CITF22-57B10) | FO |
| fw2439fw1777L | CR956437 | GGAGCTGGAACCACAAGGACTG | CR536603 (G248P-2439F13^2^) | FW | GCCTGTAGGGTCAGGACATAGAACA | gnl|ti|146870499 (G248P87333RA10.T0) | WE |
| fw2439fw1777P | CT008506 | ACACGGGCCTTCAATCAGT | CR956437 (fw2439fw1777L) | LP | CCATGCAATTAGGGACTGGT | CR933557 (fw1777b19L) | LP |
| fw1777b19L | CR933557 | TGTTCTATGTCCTGACCCTACAGGC | gnl|ti|146870499(G248P87333RA10.T0) | WE | GATATAACTGTGGGCCCCTCTGC | BX537318 (CITF22-92A6) | FO |
| c010900614rc | CR391954 | GCCAATGCAGAGTGTTCGTCTATCT | 9999.c010900614.Contig1 | SG | CACACACACATATGCACACACATGA | 9999.c010900614.Contig1 | SG |
| f96s22 | CR450365 | CTCACCGTAATGGGATTCTCATCTG | AL096853 (CITF22-96H12) | FO | AATAACGAAAATGAGAGCCGACACA | 9999.c007400748.Contig1 | SG |
| f96f65 | CR456636 | CTCACCGTAATGGGATTCTCATCTG | AL096853 (fF96H12) | FO | ACAGTTCGGAACACAGAGTGGAAAG | AL954745 (CITF22-65C6) | FO |
| c007400749 | CR380642 | TGTCGTTTCTCTTTTCATCTCTGGG | 9999.c007400749.Contig1 | SG | CAGACAGTGCTCCTGGTAGACAACA | 9999.007400748.Contig1 | SG |
| c007400748 | BX936319 | ATCACTCTTTCCCAGCTTTACGTCC | 9999.c007400748.Contig1 | SG | TTTCCCTTCACCACATCCTCACTTA | AL954745 (CITF22-65C6) | FO |
| c170L | CT00969 | AGACTCCCAACCCTCCACTT | 9999.c020502170.Contig2 | SG | GTGCCCCCAAAGCTAGGAC | 9999.c020502170.Contig2 | SG |
| aada652L | CT010407 | AAAATTCCTCTGAAACGAATACTGA | AADA01094652^3^ | CH | GAAGGTACATAAATGGCTTTGTCTT | AADA01094652^3^ | CH |
| aada652c669L | CT025869 | AAAATTCCTCTGAAACGAATACTGA | AADA01094652^3^ | CH | TCTCTGACCTGTGCCTTTTTATC | 9999.c023101669.Contig1 | SG |
| aada731b232L | CT025559 | GTCATCGGTATTCTGAGATGAACC | AADA01299731^3^ | CH | TCTTTCAATACAGGCCACATTAAGGAA | RP11-232E17 | BA |
| c004201334L | CR790389 | TGGAATGAGGAACTATTGGCTTTCA | BX649592 (G248P-80131G2^2^) | FO | CCGAGACACAGAAAGTCTCAGAAG | 9999.c004201334.Contig1 | SG |
| 36887L | CT010163 | TCACCCCATGTCCTGACTCT | AACZ02197284^1^ | CH | CCAGTTTACCCTGGGAGGTT | AACZ02197284^1^ | CH |
| c024601283L | CR932344 | GTACCAGGGGAGGGCATTATGAGT | 9999.c024601283.Contig1 | SG | CTGCACCTGGAGGCTATCTGTGAG | 9999.c024601283.Contig1 | SG |
| c283c717 | CR559946 | TATATGCAAAATAGCCTCCCATCCC | 9999.c024601283.Contig1 | SG | GGAGTTCAGTGTTGGGTTTTCTCCT | 9999.c018300717.Contig1 | SG |
| c018300717L | CR861498 | AACCATATACCTTGAGCAGCCGTTT | 9999.c018300717.Contig1 | SG | TGGAAGCCATAGGCAGAGAGTAAAA | 9999.c018300717.Contig1 | SG |
| c00717c00720L | CR932343 | AAACTGCTTCGGAGGACTGGACT | 9999.c018300717.Contig1 | SG | CTGTCTTCCTCTGCAGCTGGTGTAT | 9999.c018300720.Contig1 | SG |
| c018300720L | CR759740 | AGATGTGTGGTGCCAAGGAAAAGT | 9999.c018300720.Contig1 | SG | GCAGGATGATCTTCTCTGTGTCCTG | 9999.c018300720.Contig1 | SG |
| c720c749L | CT009622 | CTAAAACGACCTCGATCTTCCCTGT | 9999.c018300720.Contig1 | SG | TTAAGTTACCGACAGCCCCAAACTC | 9999.c009200749rc.Contig1 | SG |
| c749c575_3L | CT009623 | AACACCTCTGCAGTTTCACTTT | 9999.c009200749.Contig1 | SG | AGGAAACATATACGACGAGAAGCTA | 9999.c009200575.Contig1 | SG |
| c749aadd513L | CU041238 | GCAACACCTCTGCAGTTTCACTTT | 9999.c009200749.Contig1 | SG | GGGACAGAGACCACACAATGATTTC | AADD01175513.1 | CE |
| aadd513f116L | CU074331 | TCTCCAACTTTTATCAGCAGGGTCA | AADD01175513 | CE | TTATTTCAGATGTTAGAGCGAGCCG | BX545851 (CITF22-116F7) | FO |

Sequence source codes: SG: Chr 22 WCS from the HapMap project, YE: Sanger YAC end-sequence, CE: Celera WGS, FO: Sanger fosmid sequence, FW: Whitehead fosmid sequence, WE: Whitehead fosmid end-sequence, CH: chimp shotgun, LP: Long-PCR sequence

^1^ Chimpanzee (GenBank no. AACZ02000000). ^2^ Alternative clone name prefix: WI2- . ^3^ Chimpanzee (GenBank no. AADA01000000).
