# Supplementary material for: Finishing the finished human chromosome 22 sequence
Source: Genome Biol. 2008 May 13;9(5):R78. doi: 10.1186/gb-2008-9-5-r78 (PMC2441464; doi:10.1186/gb-2008-9-5-r78)
Supplement: Additional data file 6 — Clones identified as mapping to human chromosome 22p or 22cen. [file gb-2008-9-5-r78-S6.doc]

**Table S5**

Accession Numbers of Sequenced Clones Mapped to Chromosome 22p

AL592183

AL592188

AL590990

AL590523

AL592170

AL603926

AL591856

AL671532

BX294097

BX294002

AL929347

CT476828

CT978678

CT867976

CT867977

CT867978

CU013544

CT954328

CT963082

CU179654

CU104787

CT998553

CU462820

CU463998

CU459211

CU459201

CU459202

CU459023

CU459187

CU459125

CU442762

CU459200

CU459188

CU463998
