# Supplementary material for: Finishing the finished human chromosome 22 sequence
Source: Genome Biol. 2008 May 13;9(5):R78. doi: 10.1186/gb-2008-9-5-r78 (PMC2441464; doi:10.1186/gb-2008-9-5-r78)
Supplement: Additional data file 7 — Figures S1-S15. [file gb-2008-9-5-r78-S7.pdf]

Figure S1

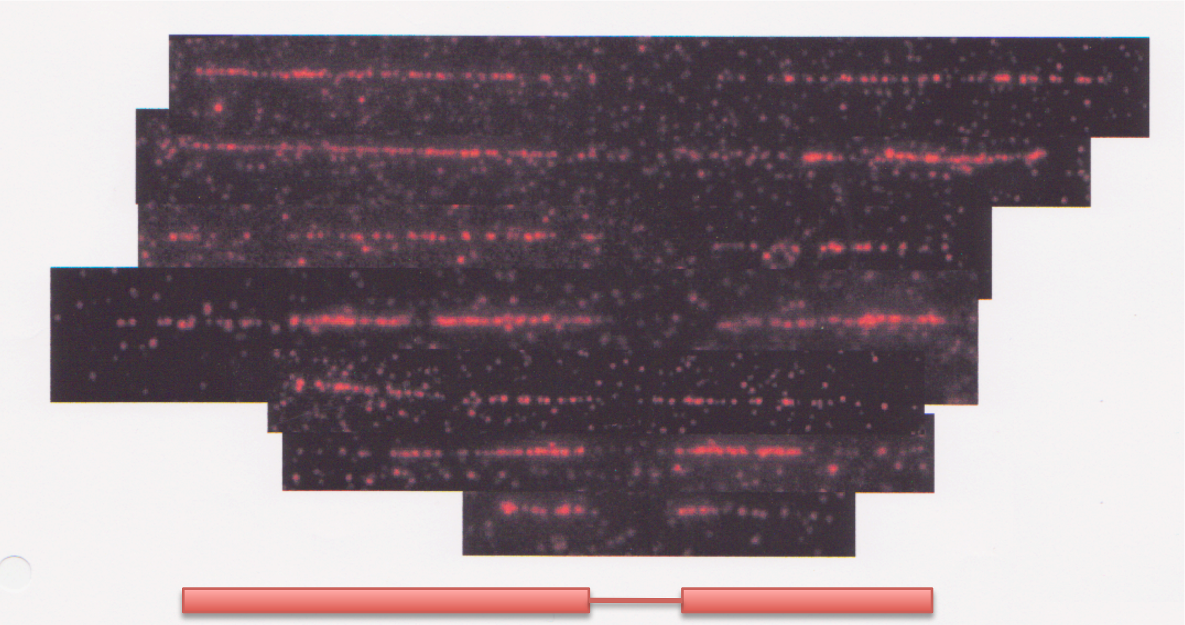

CTA-437G10

Figure S1
A deletion in BAC CTA-437G10 (AL022330) established by DNA fibre FISH analysis. The top panel shows 7 example DNA fibres from the lymphoblastoid cell line HRC575 (ECACC) hybridised with BAC CTA-437G10 and detected in red (Texas Red). Below the red boxes indicate a summary interpretation of the fibre results indicating a deletion in the BAC compared to the genomic DNA.

Figure S2

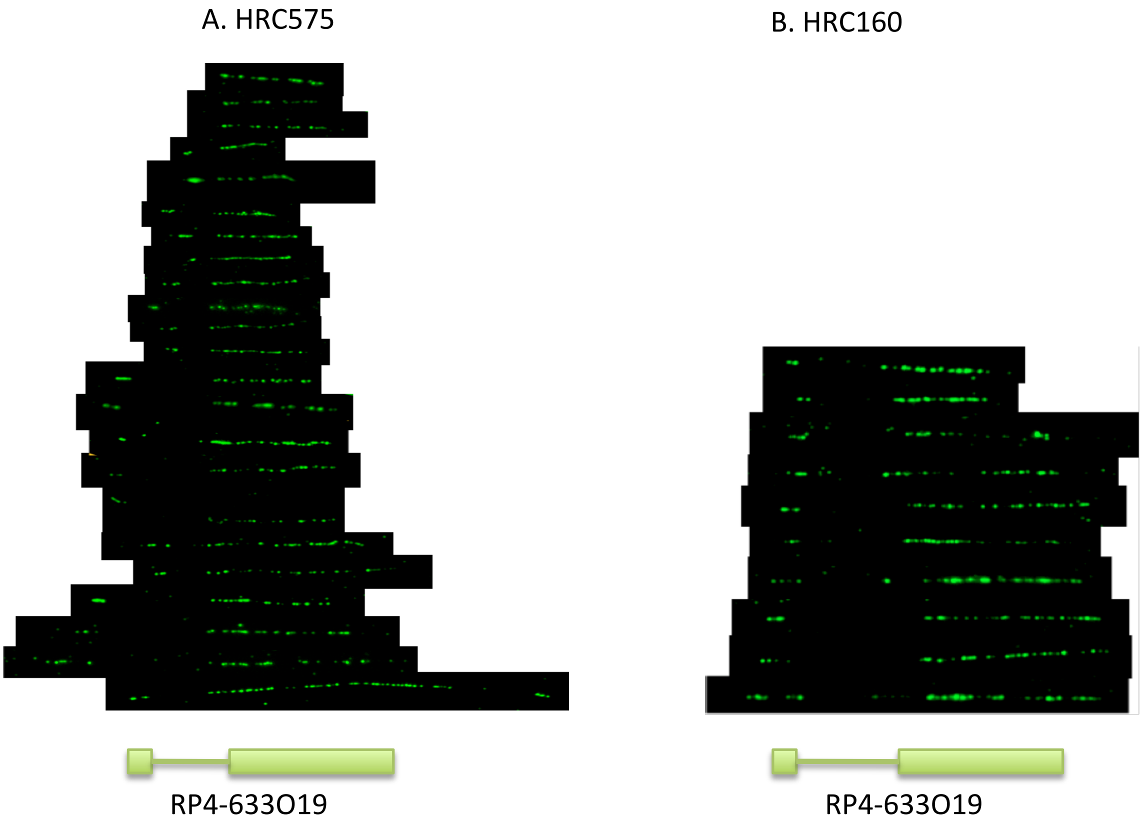

Figure S2
Deletion in PAC RP4-633O19 (AL022302) identified by fosmid end mapping, confirmed by DNA fibre FISH analysis. The top panels show multiple DNA fibres hybridised with PAC RP4-633O19 and detected in green (FITC). Genomic DNA fibres were generated from lymphoblastoid cell lines from two different individuals, HRC575 (A) and HRC160 (B). Beneath the fibres, the green boxes represent a summary interpretation of the fibre results indicating a deletion in the PAC compared to the genomic DNA.

Figure S3

A. HRC575

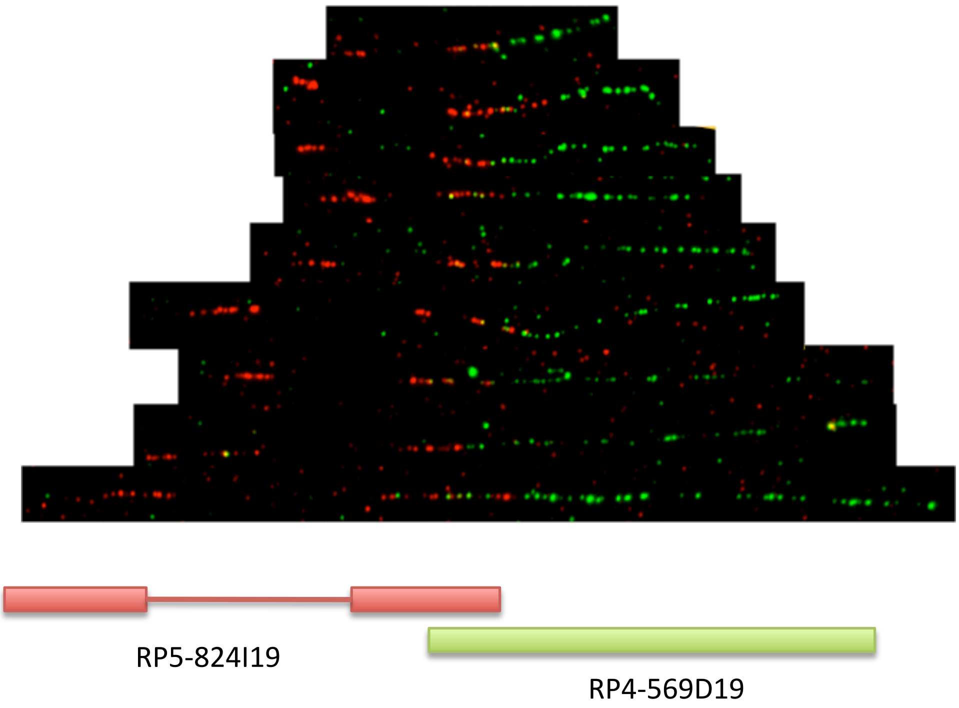

B. HRC160

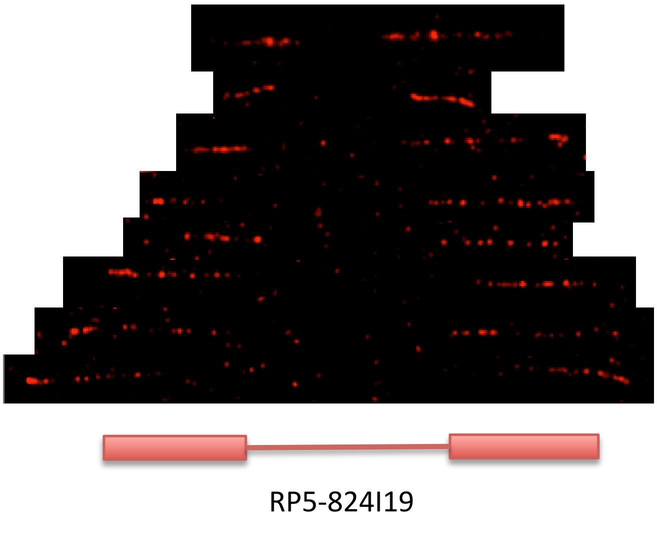

Figure S3
Deletion in RP5-824I19 (AL009049) identified by fosmid end mapping, confirmed by DNA fibre FISH analysis. A. The top panel shows multiple HRC575 genomic DNA fibres hybridised with PAC RP5-824I19 (detected in red (Texas Red)) and the neighbouring overlapping PAC RP4-569D19 (detected in green (FITC)). Beneath the fibres, the red boxes represent a summary interpretation of the fibre results indicating a deletion in the PAC compared to the genomic DNA, together with the green box indicating the overlap with PAC RP4-569D19. B. A DNA fibre FISH experiment using PAC RP5-824I19 (detected in red (Texas Red)) on HRC160 lymphoblastoid cell line DNA fibres, indicating that the deletion is common across the four haplotypes (two individuals) tested.

Figure S4 - Gap 1

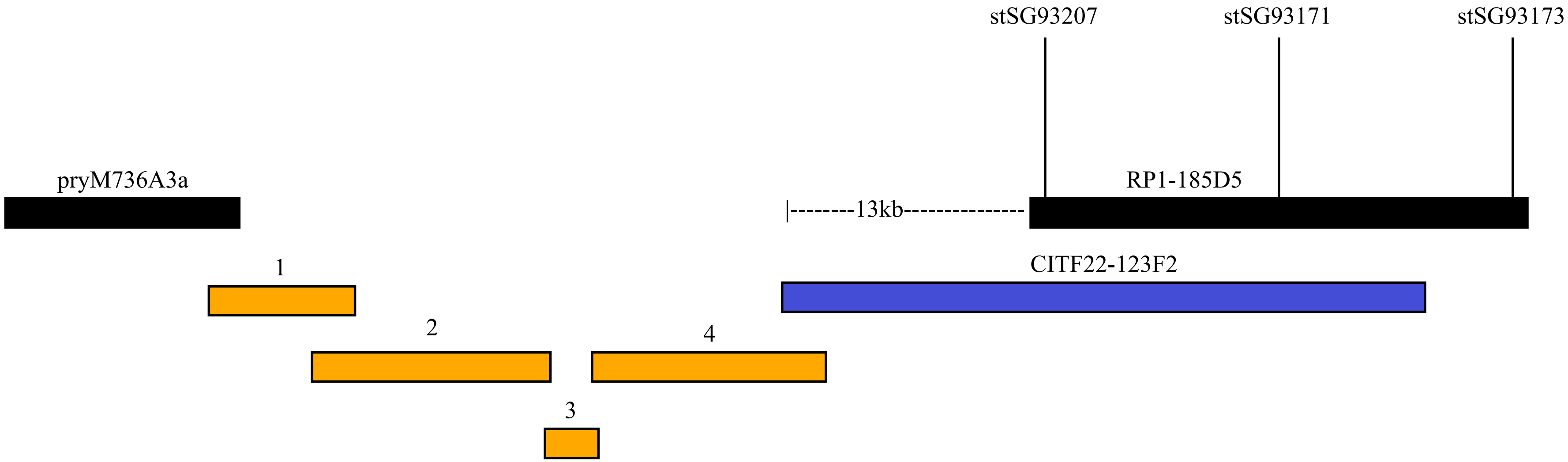

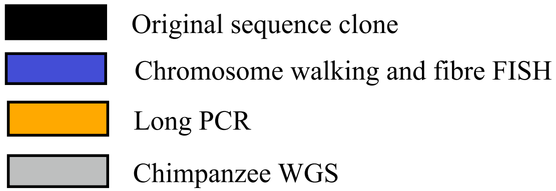

Clones

pryM736A3a	10 kb
CITF22-123F2	38 kb
RP1-185D5	24 kb

Long PCR Products

1.	c005500511	7 kb
2.	c0511c0695L	13 kb
3.	c008600695rc	2.5 kb
4.	FW80414D10l	10 kb

Figures S4-S13

Schematic views of mapping paths used to identify clones and PCR products for sequencing for 10 of the gaps as indicated. Clones and long PCR products are indicated by coloured boxes approximately to scale and shown by type according to the legend and table within each figure. Additional sources of sequence used for design of mapping reagents beyond the chromosome 22 WCS are also indicated according to the key. The figures S4-S13 should be read in conjunction with Tables S3 and S4 which list the mapping and long PCR STSs and and the sources of sequence used for their design.

Figure S5 - Gap 2

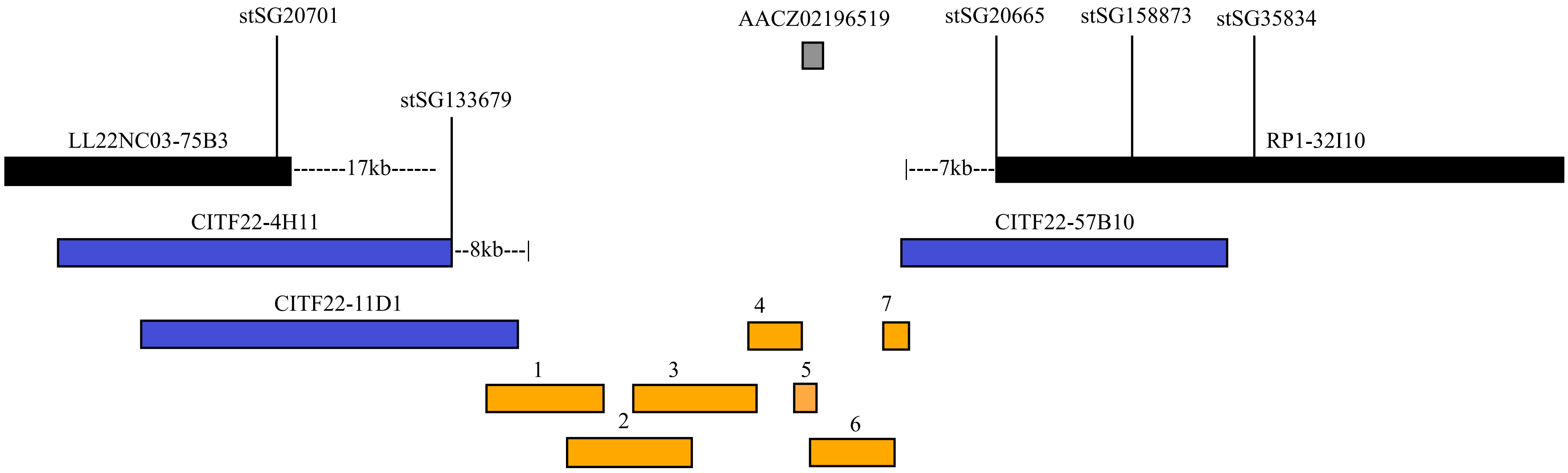

Original sequence clone
Chromosome walking and fibre FISH
Long PCR
Chimpanzee WGS

Clones

LL22NC03-75B3	40 kb
CITF22-4H11	36 kb
CITF22-11D1	37 kb
CITF22-57B10	36 kb
RP1-32I10	93 kb

Long PCR Products

1. c017600455L	6.0 kb
2. c455c658L	7.0 kb
3. c009500658L	7.0 kb
4. c658c926rcL	2.8 kb
5. c926rcL	1.0kb
6. chimpc702L	4.5kb
7. c024501702	1.3kb

Figure S6 - Gap 3

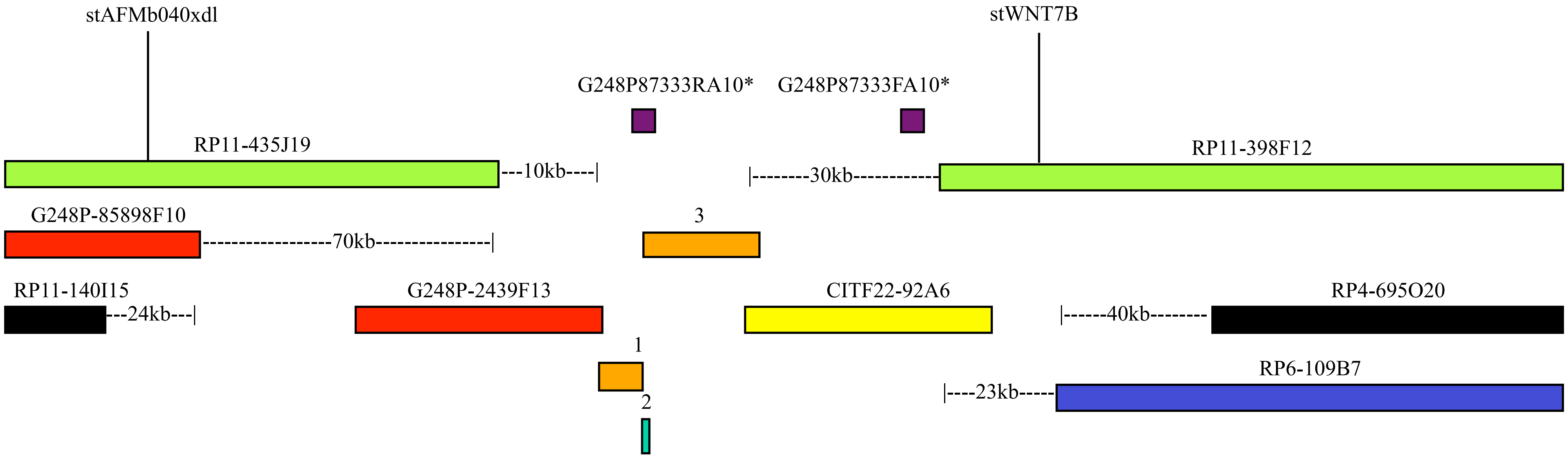

Clones

RP11-140I15	172kb
G248P-85898F10	47kb
RP11-435J19	233kb
G248P-2439F13	40kb
CITF22-92A6	42kb
RP11-398F12	215kb
RP6-109B7	215kb
RP4-695O20	93kb

Long PCR Products

1.	fw2439fw1777L	3kb
2.	fw2439fw177719P	200bp
3.	fw1777b19L	10kb

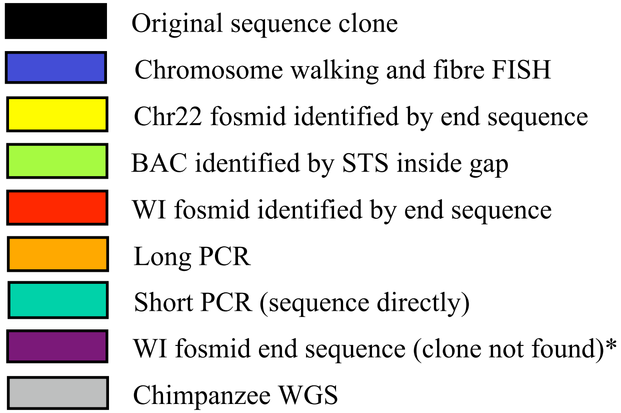

* End sequences from Fosmid G248P-1777B19, but mispick/contamination problems exist with this clone.

Figure S7 - Gap 4

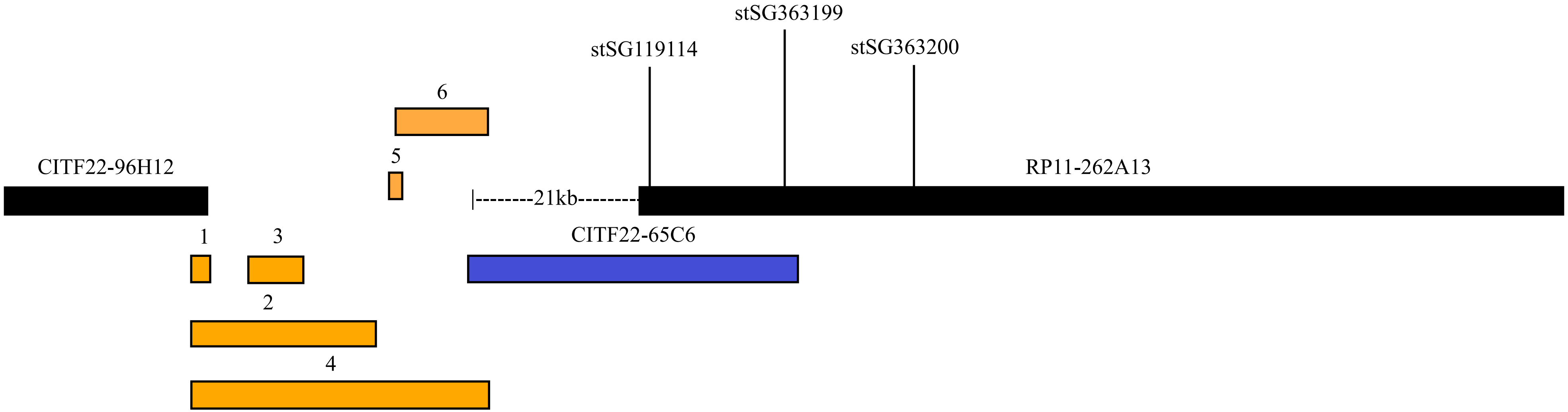

Original sequence clone
Chromosome walking and fibre FISH
Long PCR

Clones

CITF22-96H12	16kb
CITF22-65C6	39kb
RP11-262A13	128kb

Long PCR Products

1.	c010900614rc	1.4kb
2.	f96s22	15kb
3.	f96s22tandemL*[1]	5kb (single individual PCR)
4.	f96f65*[2]	23kb+
5.	c007400749	1.0kb
6.	c007400748	7kb

*[1] Sequence incorporated into f96s22 accession.

*[2] Only RHS of PCR sequence finished and submitted

Figure S8 - Gap 5

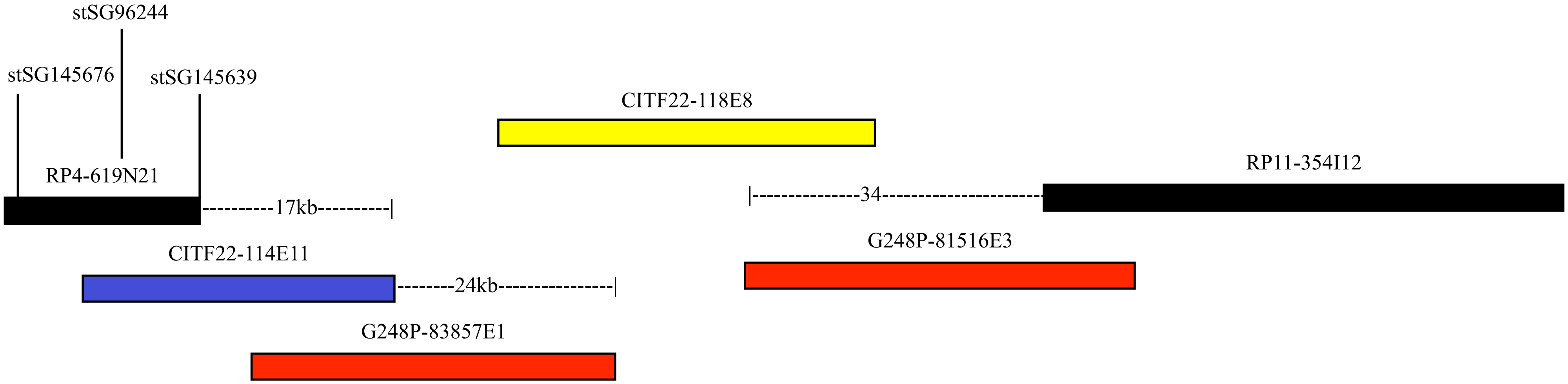

Clones

RP4-619N21	139kb
CITF22-114E11	47kb
G248P-83857E1	43kb
CITF22-118E8	40kb
G248P-81516E3	47kb
RP11-354I12	88kb

Original sequence clone
Chromosome walking and fibre FISH
Chr22 fosmid identified by end sequence
WI fosmid identified by end sequence

Figure S9 - Gap 6

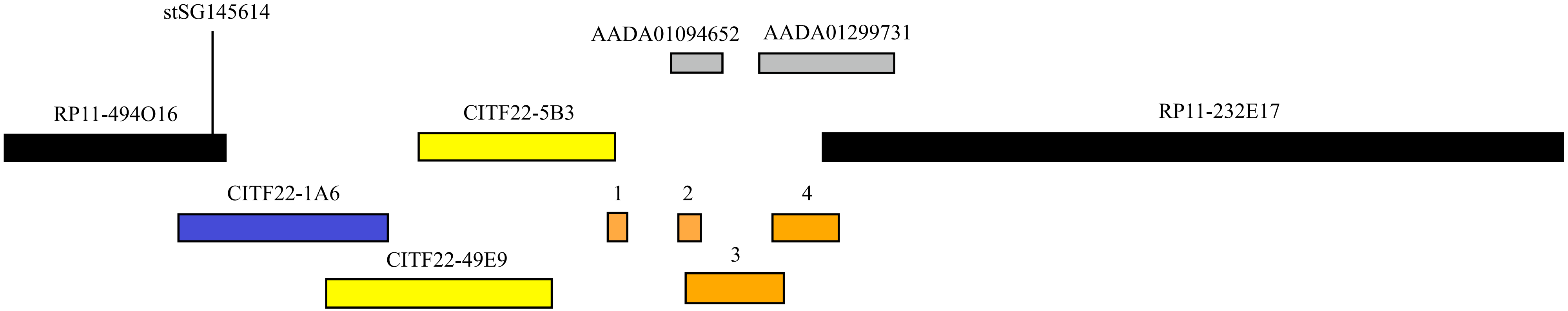

Clones

RP11-494O16	51kb
CITF22-1A6	41kb
CITF22-49E9	46kb
CITF22-5B3	43kb
RP11-232E17	170kb

Long PCR Products

1	c170L	1.3kb
2.	aada652L	1.7kb
3.	aada652c669L	6.0kb
4.	aada731b232L	4.2kb

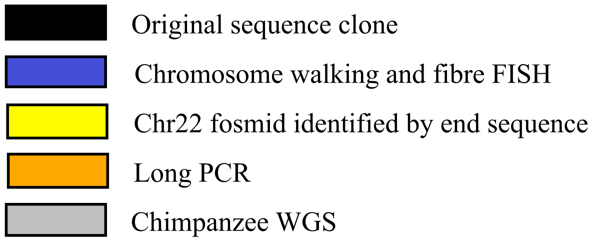

Figure S10 - Gap 7

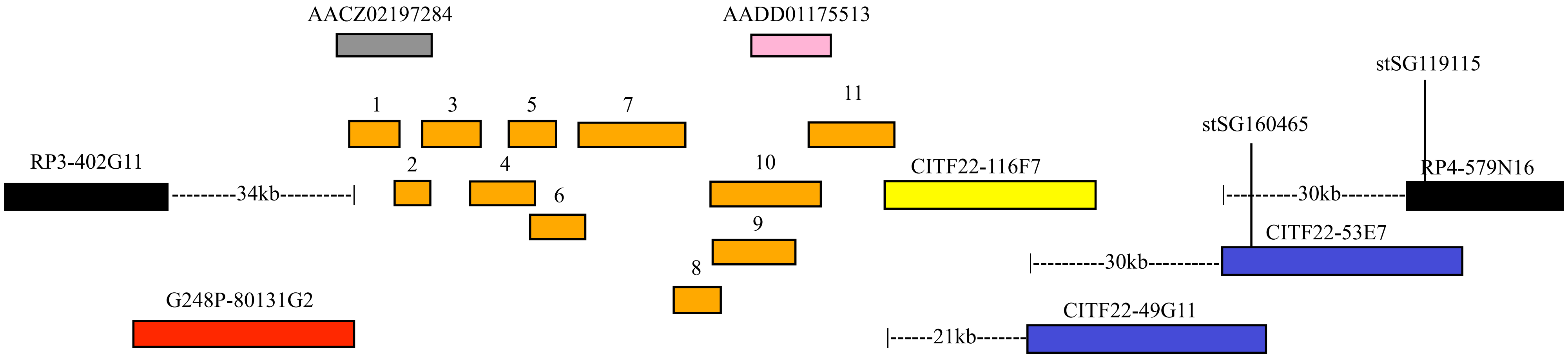

Clones

RP3-402G11	177kb
G248P-80131G2	43kb
CITF22-116F7	43kb
CITF22-49G11	46kb
CITF22-53E7	43kb
RP4-579N16	67kb

Long PCR Products

1.	c004201334L	3.1kb
2.	36887L	1.6kb
3.	c024601283L	4.4kb
4.	c283c717	4.8kb
5.	c018300717L	3.0kb
6.	c00717c00720L	3.0kb
7.	c018300720L	8.4kb
8.	c720c749L	2.7kb
9.	c749c575_3L	7.0kb
10.	c749aadd513L	9kb
11.	aadd513f116L	6kb

Original sequence clone
Chromosome walking and fibre FISH
Chr22 fosmid identified by end sequence
WI fosmid identified by end sequence
Long PCR
Celera WGSA
Chimpanzee WGS

Figure S11 - RP4-633O19 Deletion

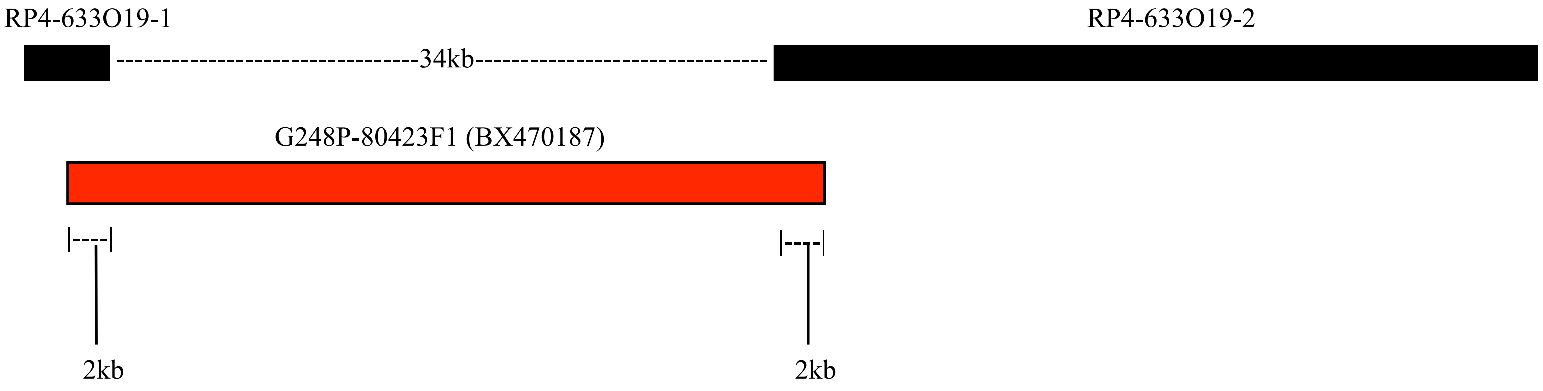

Clones

RP4-633O19-1	4kb
G248P-80423F1	38kb
RP4-633O19-2	38kb
Deletion	34kb
RP4-633O19 complete	76kb

Original sequence clone

WI fosmid identified by end sequence

Figure S12 - RP5-824I19 Deletion

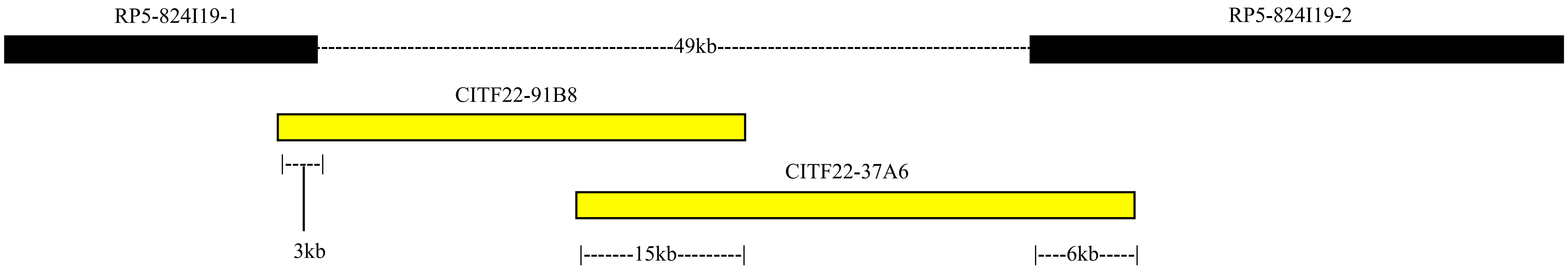

Clones

RP5-824I19-l	25kb
CITF22-91B8	35kb
CITF22-37A6	38kb
RP5-824I19-2	37kb
Deletion	49kb
RP5-824I19 complete	111kb

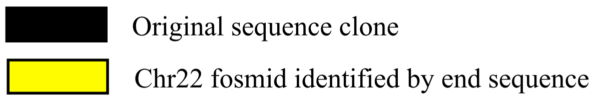

Figure S13 - Gap A

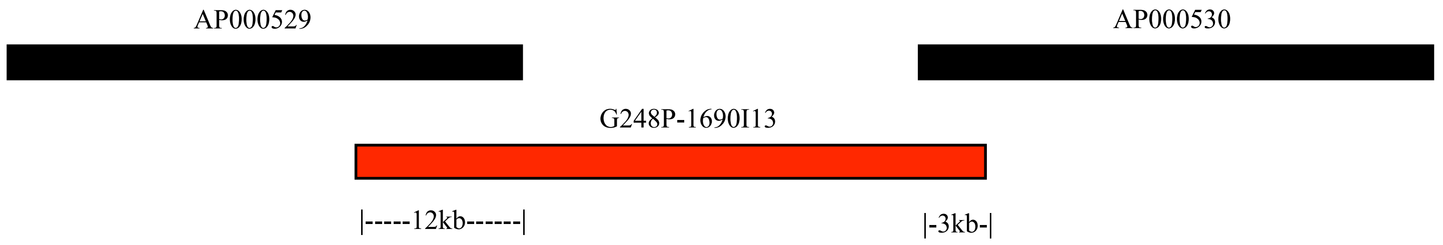

Clones

AP000529	38kb
AP000530	33kb
G248P-1690I13	22kb

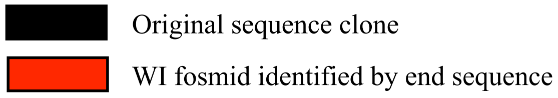

Figure S14

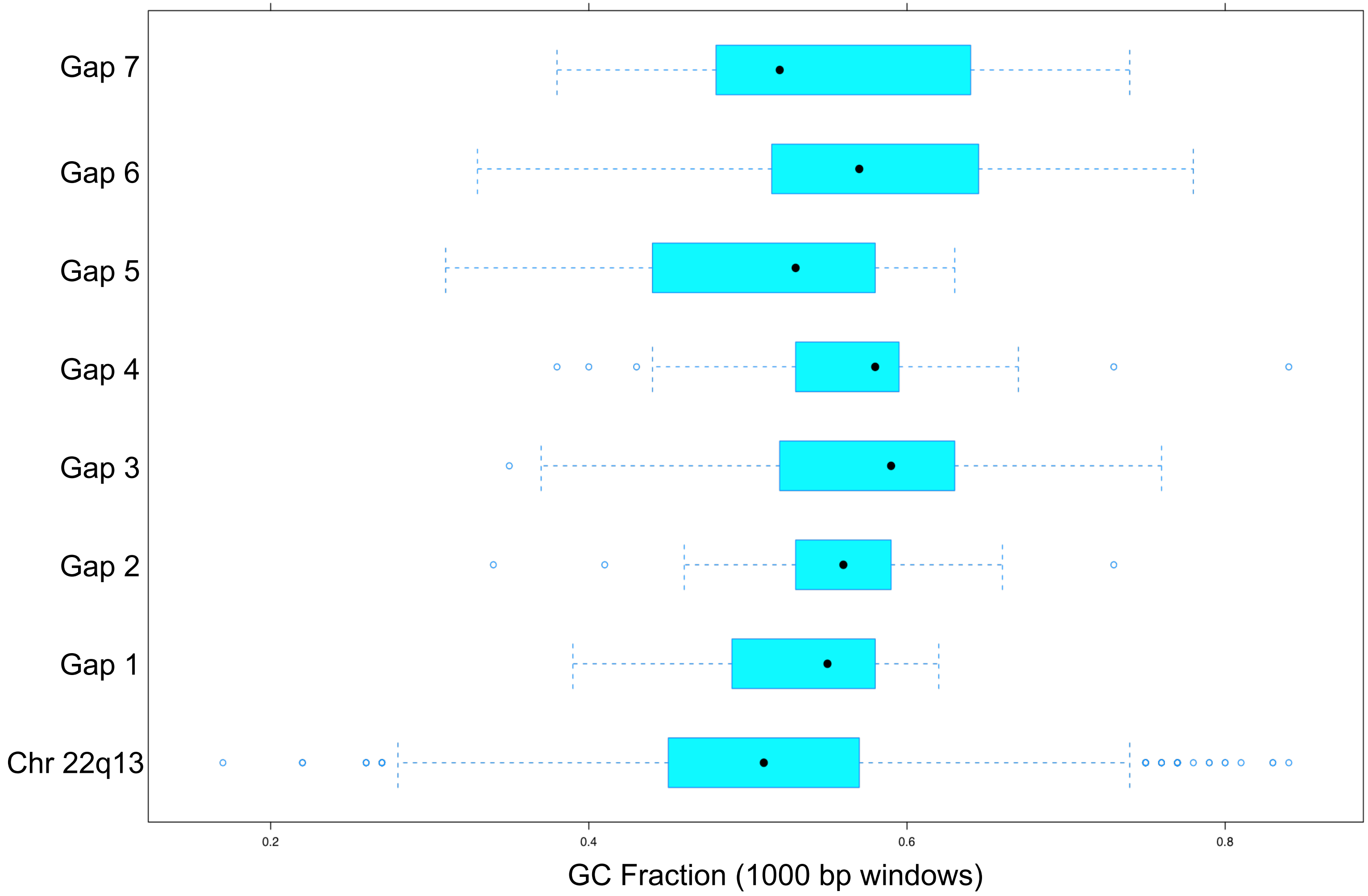

Figure S14

Box and whisker plot analysis of GC content distributions of gaps1-7 compared to the telomeric 7.3Mb of chromosome 22 (Chr_22). GC content of each of the gaps and the telomeric 7.3 Mb of chromosome 22 (22q13) was determined in 1 kb windows using custom perl scripts, and box and whisker plots of the distributions of GC fraction (x axis) for each gap and 22q13 created in R using bwplot and the default parameters for boxplot.stats. The dot shows the median value, and the box the 25th-75th quartile range. Whiskers are the limits of values within 1.5 times the interquartile range from the box.

Figure S15

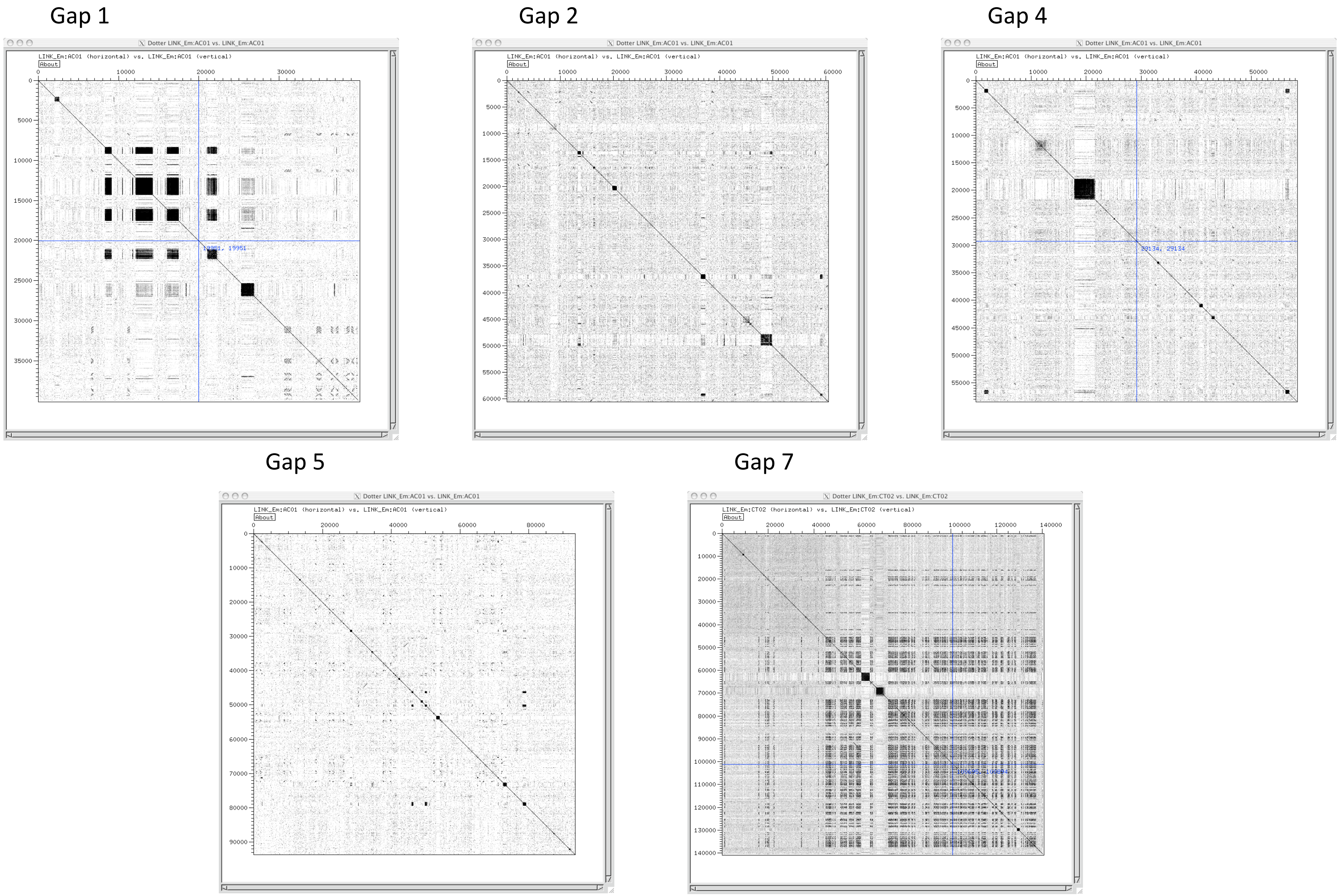

Figure S15

DNA dot plot analysis of the sequences of several gap regions reveals high density of simple tandem repeat sequences, generated using Dotter with the default settings .
